# Principles of a Non-orthogonal Optical Surface with Potential for Correction of Irregular Astigmatism

**DOI:** 10.1007/s44402-026-00068-6

**Published:** 2026-04-15

**Authors:** David A. Atchison, Arthur Ho

**Affiliations:** 1 Centre for Vision and Eye Research, Queensland University of Technology, Brisbane, Queensland Australia; 2 Brien Holden Vision Institute, Sydney, New South Wales Australia; 3 School of Optometry and Vision Science, University of New South Wales, Sydney, New South Wales Australia

**Keywords:** Irregular astigmatism, Keratoconus, Lens design, Non-orthogonal lenses

## Abstract

**Purpose:**

Non-orthogonal lenses, in which the principal meridians of the cylinder surfaces are not perpendicular to each other, can be used to improve vision in the condition of irregular astigmatism. A procedure is developed that allows raytracing and image-quality analysis with such lenses.

**Methods:**

A non-orthogonal surface was developed as a user-defined surface in the programme Ansys Zemax OpticStudio using a bi-cubic meridional mapping function. The function consists of two third-order polynomial sub-functions, each for mapping the actual ‘on surface’ meridian to an effective meridian that determines the meridional curvature for the two sectors of meridians bounded by the principal meridians. Equations were derived for partial derivatives across the surface, as Zemax requires these for raytracing through a user-defined surface.

**Results:**

Examples are shown of tangential and pupil power maps for a thin orthogonal (conventional) +5.00 DS/+2.00 DC × 180 lens and for a non-orthogonal +5.00 DS/+2.00 DC (60) × 180 lens. The orthogonal lens has principal meridians 180° and 90° while the non-orthogonal lens has principal meridians 180° and 60°. For the orthogonal lens, both maps show regular changes in power with meridional angle, and the sagittal power map is rotated by 90° relative to the tangential power map. For the non-orthogonal lens, the sagittal power pattern shows sharp changes in power. The non-orthogonal surfaces were replicated using the ‘grid sag’ surface of Zemax, and the user-defined surface was verified. Surface fitting using Zernike polynomials returned only a reasonable approximation to a non-orthogonal surface.

**Conclusion:**

A type of non-orthogonal optical surface based on two third-order polynomials is presented. Aspects of its geometrical optics properties were investigated, including confirming the non-orthogonality of its axes. This type of surface may have utility in the correction of irregular astigmatism, such as occurs in keratoconus.

Key points

- A procedure is developed that allows raytracing and image quality analysis with non-orthogonal lenses, in which the principal meridians of the cylinder surfaces are not perpendicular to each other.
- A non-orthogonal surface was developed as a user-defined surface using a bi-cubic meridional mapping process in a popular optical design programme.
- Pupil power maps were used to compare tangential and sagittal power patterns for a non-orthogonal lens and its equivalent orthogonal lens.

## Introduction

Keratoconus is an eye condition characterised by progressive, ectatic changes in the cornea. While at early stages of severity, vision correction using ophthalmic devices such as spectacles or contact lenses may be adequate, the presence of irregular astigmatism leading to higher-order aberrations tends to degrade even best-corrected vision [1]. For this reason, clinicians are constantly seeking new solutions for providing vision for keratoconus conditions.

Hulpus and colleagues [2] described the use of “non-orthogonal” cylinder trial lenses in refraction for patients with keratoconus. The powers went out to −3.00 in 0.50 D steps. These trial lenses had the differences between principal meridians at less (and greater than) than 90°, with a smaller angle difference of the lenses being 60–85° in 5° steps. Using modifications to refraction techniques, the authors were able to improve visual acuity and subjective evaluation with the optimal non-orthogonal correction in the majority of participants. Most smaller angle differences for optimum refraction were 80° and 85°.

In an earlier paper [3], the same authors indicated that the optics of keratoconic eyes might not be fully captured by Zernike polynomials. This means that so-called “irregular” astigmatism may not be attributable purely to comatic higher-order aberration. Also, the authors indicated that non-orthogonal lenses may not necessarily be appropriate beyond a small central region.

Some design work on non-orthogonal lenses was presented in the earlier paper [3], but it is not clear how this was applied to the cylinder trial lenses in the clinical trial [2]. The main purpose of this work is to develop non-orthogonal lens surfaces that can be used for designing improved ophthalmic lenses in conditions such as keratoconus. Before doing this, we will show how the power of non-orthogonal lenses can be represented and the transpositions between positive and negative cylinder forms for such lenses.

## Power Representation of Non-orthogonal Cylinders

**Fig. 1:**
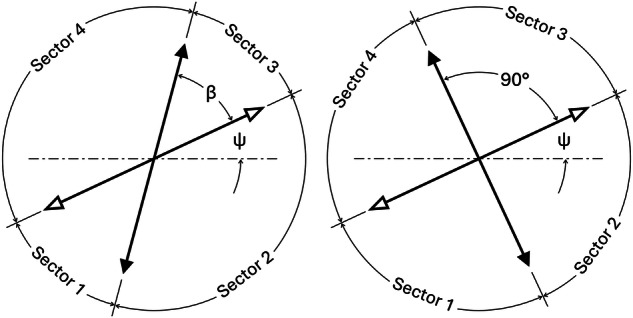
The four sectors of a non-orthogonal cylinder (left) and their corresponding sectors for a conventional cylinder (right). The solid black arrow indicates the cylinder meridian, while the hollow arrow indicates the meridian for the base power.

**Fig. 2:**
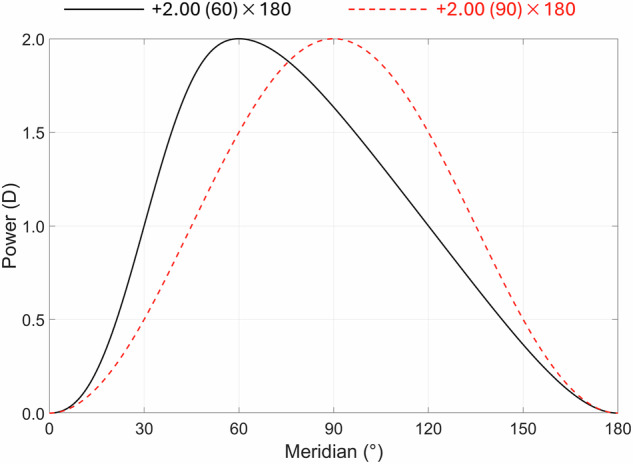
Power as a function of meridian for the non-orthogonal cylinder +2 (60) × 180 and orthogonal (conventional) cylinder +2 (90) × 180. Relating to Fig. 1, *ψ* is 180° for both cylinders, while *β* is 60° and 90° for the non-orthogonal cylinder and orthogonal cylinder, respectively.

We present here a simple radial (also tangential or meridional) power representation of a non-orthogonal cylinder lens, given as *F*(*β*) × *ψ* [2], where principal meridians are *ψ* and *ψ* + *β*. *β* is >0 and <180°. For an orthogonal, i.e., conventional, cylinder lens, *β* is 90°. The front surface is flat, and the back surface tangential power is represented as a function of meridian ${\theta }_{{{m}}}$ (see Appendix) by relating the meridian to an equivalent meridional power in the corresponding sector of a conventional cylinder. Four sectors are used, of which three are needed within the 0–180° range for any *ψ*, *β* combination. Table 1 and Fig. 1 define the meridional positions of these sectors for a non-orthogonal cylinder and their corresponding sectors on a conventional cylinder. Figure 2 shows the radial power as a function of meridian for +2.00 (60) × 180 and +2.00 (90) × 180 surfaces; these are the powers that would be measured with a Geneva lens measure as it is rotated about the optical axis.

**Table 1:** The meridional positions of the four sectors used in determining the meridional power of a non-orthogonal cylinder and their corresponding sectors for a conventional cylinder.

Sector	Non-orthogonal cylinder	Conventional cylinder
1	$\psi -180\le {\theta }_{{{\rm{m}}}} < \psi -180+\beta$	$\psi -180\le {\theta }_{{{\rm{m}}}} < \psi -90$
2	$\psi -180+\beta \le {\theta }_{{{\rm{m}}}} < \psi$	$\psi -90\le {\theta }_{{{\rm{m}}}} < \psi$
3	$\psi \le {\theta }_{{{\rm{m}}}} < \psi +\beta$	$\psi \le {\theta }_{{{\rm{m}}}} < \psi +90$
4	$\psi +\beta \le {\theta }_{{{\rm{m}}}} < \psi +180$	$\psi +90\le {\theta }_{{{\rm{m}}}} < \psi +180$

Principal meridians are *ψ* and *ψ* + *β*, and the general meridian is *θ*_m_.

## Sphero-cylindrical Transposition with Non-orthogonal Cylinders

For completeness, the rules for transposing between positive and negative cylinder forms are:

- New sphere = original sphere + original cylinder;
- New cylinder = opposite of original cylinder;
- New primary axis = original primary axis + original $\upbeta$ or original primary axis + original $\upbeta$ –180° [using whichever of the right-hand side parts keeps the new primary axis in the range 0–180°];
- New $$\upbeta $$ = 180 − original $$\upbeta $$.

Using symbols, these equations are:

1 $${S}_{{{\rm{new}}}}={S}_{{{\rm{orig}}}}+{C}_{{{\rm{orig}}}}$$

2 $${C}_{{{\rm{new}}}}=-{C}_{{{\rm{orig}}}}$$

3 $${\psi }_{{{\rm{new}}}}={\psi }_{{{\rm{orig}}}}+{\upbeta }_{{{\rm{orig}}}}\,{{\rm{or}}}\,{\psi }_{{{\rm{orig}}}}+{\upbeta }_{{{\rm{orig}}}}-{180}^{\circ }$$

This equation can be given also as

3a $${\psi }_{{{\rm{new}}}}=({\psi }_{{{\rm{orig}}}}+{\upbeta }_{{{\rm{orig}}}})\,{{modulo}}\,{180}^{\circ }$$

which means that *ψ*_new_ is the remainder from (*ψ*_orig_ + *β*_orig_)/180°

4 $${\upbeta }_{{{\rm{new}}}}=180-{\upbeta }_{{{\rm{orig}}}}$$

For conventional cylinders, these become equivalent to the procedures given in texts such as Bennett [4] and Jalie [5]. For a conventional cylinder with *β*_orig_ = 90°, Eqs. (1) and (2) are applied as above. Equations (3) and (3a) become, respectively,

*ψ*_new_ = *ψ*_orig_ ± 90° (using the sign keeping *ψ*_new_ in the range 0–180°)

and

$${\psi }_{{{\rm{new}}}}=({\psi }_{{{\rm{orig}}}}+{90}^{\circ })\,{{\rm{modulo}}}\,{180}^{\circ }$$

Equation (4) becomes moot.

Table 2 shows three examples of sphero-cylinder transpositions. The first is a conventional (orthogonal) case so that *β*_orig_ and *β*_new_ are 90°, and the second has the same values as the first example, except that *β*_orig_ is 60°. The first two examples start with positive cylinders, and the third example starts with a negative cylinder.

**Table 2:** Three examples of sphero-cylinder transpositions with non-orthogonal cylinders.

Example	1. Plano /+2.00 (90) × 180	2. Plano /+2.00 (60) × 180	3. +3.00 /−4.00 (50) × 120
Eq. (1): *S*_new_	0.00 + 2.00 = +2.00	0.00 + 2.00 = +2.00	+3.00+(–4.00) = –1.00
Eq. (2): *C*_new_	–(+2.00) = –2.00	–(+2.00) = –2.00	–(–4.00) = +4.00
Eq. (3): *ψ*_new_	(180° + 90°) modulo 180° = 90°	(180° + 60°) modulo 180° = 60°	(120° + 50°) modulo 180° = 170°
Eq. (4): *β*_new_	180°–90° = 90°	180°–50° = 130°	180°–50° = 130°
Transposed form	+2.00/–2.00 (90) × 90	+2.00/–2.00 (120) × 60	–1.00/+4.00 (130) × 170

Values of *ψ* and *β* are: Example 1, *ψ* = 180°, *β* = 90°; Example 2, *ψ* = 180°, *β* = 60°; Example 3, *ψ* = 120°, *β* = 50°.

## Methods

### Meridian Mapping Function

An outline of the method of construction of the non-orthogonal surface is given here. Fuller details are given in the Appendix.

In a conventional (orthogonal) cylindrical surface, the angles between the two principal meridians are perpendicular. A non-orthogonal surface, wherein the meridional angles between principal meridians are not 90°, may be constructed by relating (i.e., mathematically mapping) the meridians on its surface to corresponding meridians on a conventional cylindrical surface. As an analogy, a conventional cylindrical surface can be thought of as an umbrella. Each rib of the umbrella represents a meridian. The arc of each rib represents a meridional curvature. A non-orthogonal surface is analogous to angularly repositioning the ribs (by rotation about the umbrella shaft) while retaining the curvature of each. That is, for each actual/physical meridian on the non-orthogonal surface, a corresponding meridian on a conventional meridian is determined using a suitable mapping function that defines the meridional curvature of the non-orthogonal surface along that physical meridian.

That is, a given meridional angle *θ*_m_ on the non-orthogonal surface is mapped to an equivalent meridional angle *θ*_e_ on a conventional cylindrical surface by some mapping function:

5 $${\theta }_{{{\rm{e}}}}=F({\theta }_{{{m}}})$$

where

*θ*_m_ is the angle for the actual meridian on the non-orthogonal surface, and

*θ*_e_ is the equivalent angle on a conventional cylindrical surface that defines the meridional curvature

The curvature (and hence also tangential power with refractive indices known) for a given meridian *θ*_m_ on the non-orthogonal surface can then be determined from:

6 $$C({\theta }_{{{m}}})={C}_{{{{\rm{p}}}}}+({C}_{{{{\rm{s}}}}}\,{\textstyle {{-}}}\,{C}_{{{{\rm{p}}}}}){\sin }^{2}({\theta }_{{{{\rm{e}}}}})$$

where

*C* is meridional curvature along *θ*_m_.

*C*_p_ is curvature along the primary meridian (assumed to be 180°)

*C*_s_ is curvature along the secondary meridian.

Once the meridional curvature is determined, the surface sag (*z*) can be determined at any point along a meridian using a standard sag formula. For example, for a non-orthogonal surface whose meridional profile is definable as a circular arc, a suitable formula for calculating surface sag at any point along a given meridian is

7 $$z({\theta }_{{{m}}},r)=\frac{C({\theta }_{{{m}}}){r}^{2}}{1+\sqrt{1-{(C({\theta }_{{{m}}}))}^{2}{r}^{2}}}$$

where

*θ*_m_ is the meridional angle that the surface point lies on,

*C*(*θ*_m_) is the curvature along meridian *θ*_m_ as previously defined,

*r* is the radial position of the surface point from the lens axis, and

*z* is the sag height of the surface point.

A bi-cubic mapping function was developed for relating the actual meridional angle to its corresponding equivalent meridional angle.

The bi-cubic mapping function consists of two third-order polynomial sub-functions, each for mapping the actual ‘on surface’ meridian *θ*_m_ to an effective meridian (*θ*_e_) that determines the meridional curvature for the two sectors of meridians bounded by the principal meridians. Each sub-function has the form:

8 $${\theta }_{{{\rm{e}}}}={b}_{0}+{b}_{1}{\theta }_{{{m}}}+{b}_{2}{\theta }_{{{m}}}^{2}+{b}_{3}{\theta }_{{{m}}}^{3}$$

where

${b}_{i}$ are the coefficients for the third-order polynomial.

The coefficients are determined (see Appendix) so that the third-order polynomial sub-functions are constrained to ensure C1-continuity (i.e., common position and tangent) at their junctions, as well as unity slope at the meridional angles for the two principal meridians.

Equations were derived for $\frac{{{d}}z}{{{d}}{\theta }_{{{m}}}}$ and $\frac{{{d}}z}{{{d}}r}$ as Zemax requires these partial derivatives for raytracing through a user-defined surface.

**Fig. 3:**
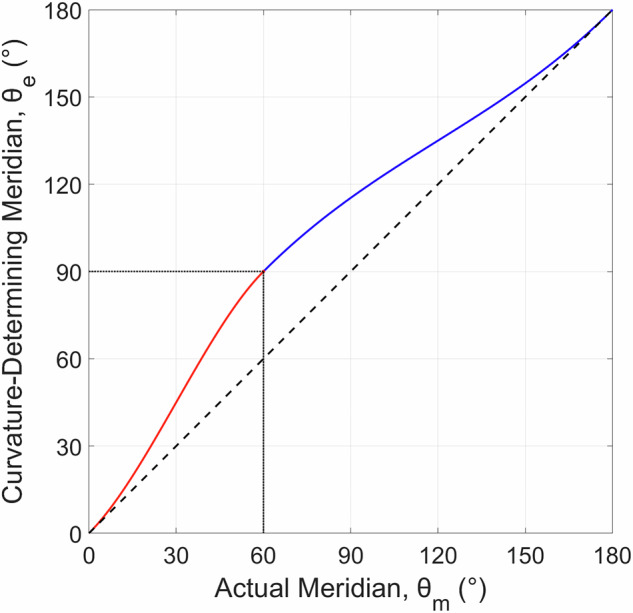
The bi-cubic mapping function relating the curvature-determining meridional angle *θ*_*e*_ to the actual meridional angle *θ*_m_, consisting of the lower sub-function (red) and upper sub-function (blue). Example shown is for the case *F*(60) × 180. The 1:1 dashed line represents the mapping for a conventional cylinder.

An example of the bi-cubic function mapping actual to equivalent meridional angles is shown in Fig. 3.

### Test Lenses and Analyses

We analysed the optics of a lens that incorporates a bi-cubic non-orthogonal surface and of a conventional (orthogonal) cylindrical lens with the same nominal meridional curvatures and tangential powers along their principal meridians. Their prescriptions are given in Table 3.

**Table 3:** Prescriptions with axis and meridional parameters of the non-orthogonal and orthogonal lenses tested.

Test lenses			Orthogonal	Non-orthogonal
Prescription			$\frac{+5.00}{+2.00\,\times 180}$	$\frac{+5.00}{+2.00\,(60)\times 180}$
Front surface	Primary	Power (D)	+5.00	+5.00
		Axis	90°	90°
		Meridian	180°	180°
	Secondary	Power (D)	+7.00	+7.00
		Axis	180°	120°
		Meridian	90°	60°
Refractive index			1.50	1.50
Back surface	Power (D)		0.00	0.00
Overall lens diameter (mm)			20	20
Aperture diameter (mm)			5	5

The bi-cubic non-orthogonal surface was coded as a user-defined surface in the programme Ansys Zemax OpticStudio (64-bit, version 2025 R2.02, ansys.com/products/optics/ansys-zemax-opticstudio). Each of the test lenses was modelled in Zemax using the user-defined surface. Raytracing was conducted on each lens model to produce tangential and sagittal power maps across the pupil. Spot diagrams for the orthogonal and non-orthogonal lenses were computed with an aperture diameter of 5 mm at the paraxial focal positions of their principal powers and at an intermediate plane approximately mid-linear distance between the two paraxial focal positions. For each spot diagram, the points of intercept of 1519 rays (22 hexapolar rings) were plotted, providing an estimate of the blur spot on the transverse plane at the intercept position.

For the non-orthogonal lens, a video of a through-focus spot between its paraxial focal positions was created. Spot diagrams were computed with Zemax at image positions from 200 mm (+5 D dioptric equivalent) to 142.86 mm (+7 D) in 0.01 D steps. The collection of 201 images was composited to produce the video.

To verify the correct coding of the user-defined surface, the non-orthogonal lens was also modelled in Zemax using the in-built “grid sag” surface type. In this model, the sagittal heights of the non-orthogonal lens surface were computed for points over an *x*, *y* grid with 0.1 mm intervals using Eqs. (A5) and (A6) (see Appendix).

We also explored the feasibility of replicating a bi-cubic non-orthogonal surface using Zernike polynomials. The pupil power map (Zemax operand POWP) of the non-orthogonal lens modelled with the user-defined surface was used to construct a merit function for optimisation in Zemax. The merit function consisted of pupil power values for 19 rings and 36 spokes of ray intercept points. The computed pupil power values were then set as target values in the merit function using a set of POWP operands (pupil power at specified points). The non-orthogonal surface was replaced by a Zemax Zernike standard sag surface. Incrementally, up to 171 coefficients for the Zernike surface were allowed to vary over a series of optimisation to fit a Zernike surface that matches the original non-orthogonal surface in terms of pupil powers.

Sagittal and tangential pupil power maps were computed for the grid sag lens model and the fitted Zernike lens model.

Due to the right-hand coordinate system followed by Zemax, all graphical results (pupil power maps and spot diagrams) presented here have been reflected about a vertical axis to comply with the ophthalmic convention of positive X pointing to horizontal right when looking towards the wearer’s eye and positive angles incrementing counterclockwise from the horizontal axis.

## Results

**Fig. 4:**
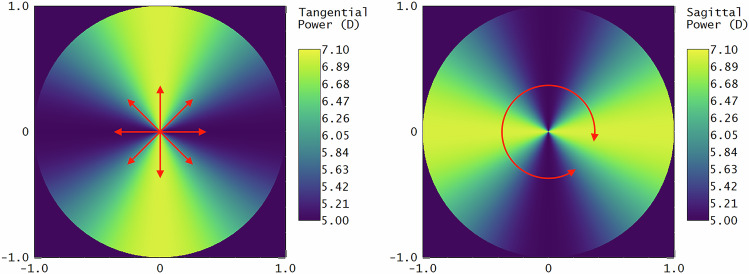
Pupil power maps for the orthogonal +5.00/+2.00 × 180 lens. (Left) Tangential with red arrows indicating that powers are measured radially, (right) sagittal with red arc and arrows indicating that powers are measured circumferentially.

Figure 4 shows tangential (radial) and sagittal (circumferential) pupil power maps for the orthogonal (+5.00/+2.00 × 180) lens. “Paraxial” tangential power varies from +5.0 D along 180° to +7.00 D along 90°. The sagittal power pattern is that of the tangential power but rotated by 90°.

**Fig. 5:**
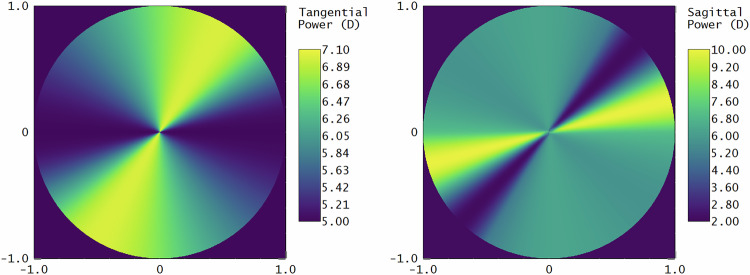
Power pupil maps for the +5.00/+2.00 (60)×180 non-orthogonal lens. (Left) Tangential and (right) sagittal. Note the difference in scales.

Figure 5 shows tangential and sagittal pupil power maps for the non-orthogonal (+5.00/+2.00 (60)×180) lens. The paraxial tangential power varies from +5.0 D along 180° to +7.0 D along 60°. The sagittal power pattern is completely different from that in Fig. 4: power varies from +2.5 D along 60° to +9.5 D along 180° and there are sharp changes of power between 60° and 180°.

**Fig. 6:**
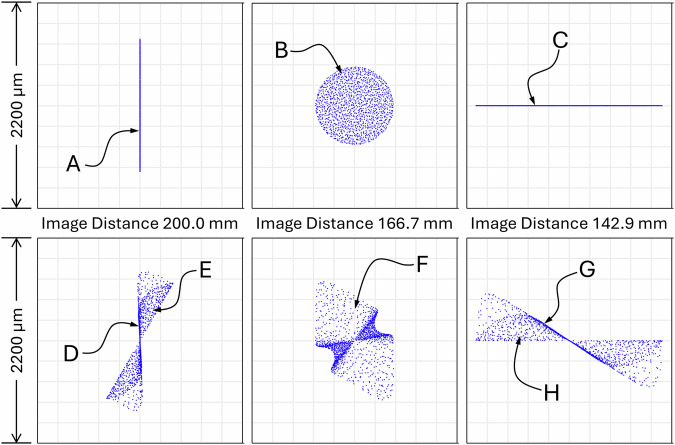
Spot diagrams for a +5.00/+2.00 × 180 orthogonal lens (top) and a +5.00/+2.00 (60)×180 non-orthogonal lens (bottom) at the paraxial focal position for 5 D (left), an intermediate position of 6 D (middle) and at the paraxial focal position for 7 D (right). For the non-orthogonal lens, note the line foci oriented along 90° (D) and 150° (G) at the 5 and 7 D focal positions, respectively. Fan-like aberration in the form of ray deviations in the circumferential direction (e.g., E and H) can be seen.

Spot diagrams for the orthogonal and non-orthogonal lenses at the paraxial focal positions of the principal powers and at an intermediate plane are shown in Fig. 6. The spot diagrams for the orthogonal lens show line foci at along 90° (A in Fig. 6) and 180° (C) for the 5 and 7 D cylinder powers, respectively. The spot diagrams for the non-orthogonal lens confirm the non-orthogonality, with line foci along 90° (D) and 150° (G) for the 5 and 7 D cylinder powers, respectively, but these are not as distinct as for the orthogonal lens due to the presence of fan-like aberration adjoining the line foci (e.g., E and H).

A video, with each frame annotated to indicate the image position and dioptric equivalent, showing through-focus spot imageries for the non-orthogonal lens, is available as a supplementary file.

**Fig. 7:**
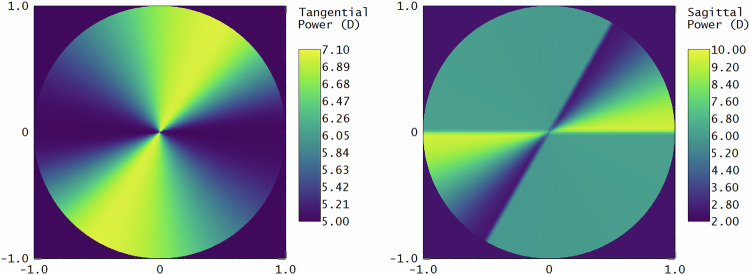
Pupil power maps for the grid-sag lens to check results for the non-orthogonal lens. (Left) Tangential and (right) sagittal. Note the difference in scales.

The pupil power maps for the non-orthogonal lens modelled using grid sag are shown in Fig. 7, exhibiting good matches to those in Fig. 5. This gave support for the accuracy of the non-orthogonal surface modelled by the user-defined surface.

**Fig. 8:**
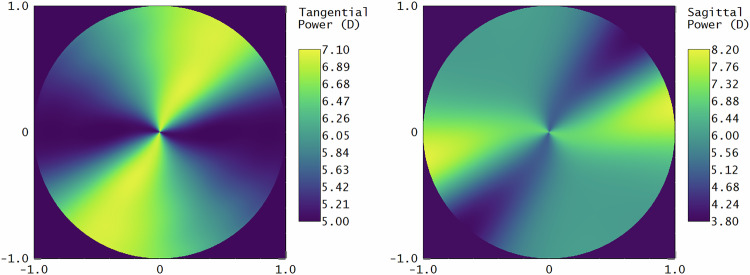
Power pupil maps for Zernike-approximated lens (left) tangential, (right) sagittal. The sagittal power map differs substantially from those of the user-defined surface (Fig. 5) and the grid-sag surface (Fig. 7). Note the difference in scales.

To replicate the non-orthogonal lens using the Zernike surface, a minimum of 105 coefficients (up to and including 13 radial orders and ±13 azimuthal frequencies) were found to be required to achieve a loosely satisfactory approximation to the surface modelled by the user-defined surface (Fig. 8). Increasing both radial order and azimuthal frequency returned decreasing and only marginal improvement.

## Discussion

A procedure has been determined to allow raytracing with non-orthogonal lenses, in which the principal meridians of the principal surfaces are not perpendicular to each other. This was verified by the spot diagrams at the focal positions for the two meridional powers (Fig. 6). In addition, the form and sphero-cylinder transpositions of such lenses were described.

A lens utilising the bi-cubic non-orthogonal surface produces its line foci at the same axial positions as the corresponding orthogonal cylindrical lens. As seen in the spot diagrams in Fig. 6, it differs from orthogonal lenses in that the angles of the line foci are not perpendicular—i.e., non-orthogonal.

It should be noted that between the two line foci (i.e., the insterval of Sturm), the non-orthogonal lens does not exhibit a true circle (or disk) of least confusion. As can be seen in Fig. 6, at the mid-dioptric position of 6 D (166.7 mm image position), the shape of the blur spot is not circular (F in Fig. 6). Further, as can be seen in the supplementary through-focus spot video, the shape of the blur spot does not transition in a predictable way between one line focus to the other as does an orthogonal lens. Rather, the through-focus blur spot morphs through several non-intuitive shapes. Moreover, during the through-focus transition, there are instances where the blur spot appears to include a line focus at different angles. In the through-focus spot video, one apparent line focus can be seen when the image plane is positioned at about 171.82 mm (5.82 D) with the focal line lying along ~60°). Another apparent line focus occurs at about 161.81 mm (6.18 D) and lies along ~3°. The impact this may have on vision for the lens wearer would need to be investigated in future studies.

Replicating the non-orthogonal surface with Zernike polynomials returned only loosely satisfactory results. While theoretically, with a sufficient number of coefficients, it should be possible to model any continuous surface with the Zernike set, in the case of the bi-cubic non-orthogonal, an impractical number of coefficients was required to achieve even a moderate approximation. The cause is due to the second derivative discontinuity of the two sub-functions where they meet. This results in a discontinuity of the sagittal power meridionally at the principal meridians (compare the sagittal power maps for Figs. 5, 7 and 8), thus requiring a large number of azimuthal frequency terms to approximate.

For the same reason, this discontinuity, whereby the sagittal powers differ on either side of the principal meridians, introduces a circumferentially asymmetrical spread of ray intercepts on either side of the line foci. This can be seen in the spot diagrams (Fig. 6), increasing in severity with ray height.

**Fig. 9:**
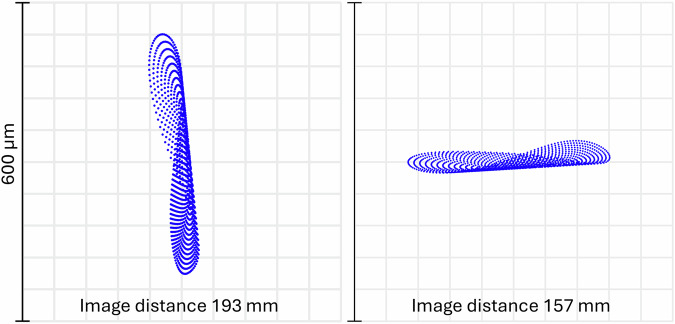
Off-axis spot diagrams for the lens of Figs. 5 and 6 at the estimated image positions for the line foci when a 2 mm diameter aperture is decentred 2 mm down and to the right of the lens geometrical axis. The line foci are close to being orthogonal.

An additional observation was made of the off-axis optics of this non-orthogonal surface. Figure 9 shows off-axis spot diagrams for the lens of Figs. 5 and 6. The aperture stop has been reduced to 2 mm diameter and decentred by 2 mm along the 315° meridian (i.e., down and to the right when looking in the direction of light) from the geometrical axis of the non-orthogonal surface. The spot diagrams were computed with the image plane located at the estimated positions for line foci. Although substantially aberrated, the two line foci are close to being orthogonal. This indicates that in certain off-axis situations, a non-orthogonal lens may behave like a conventional cylinder lens. The axes of the off-axis line foci change depending on the position and size of the aperture. This observation is of relevance to devices such as spectacle lenses, and further investigation of the off-axis behaviour of non-orthogonal lenses is warranted.

For simplicity of derivation and computation, the equations developed for the calculation of the bi-cubic non-orthogonal surface (see Appendix) assumed the primary meridian is along 180°. Analysing lens prescriptions for which the primary axis is not 180° can be undertaken by introducing a rotation of the coordinate system around the lens axis by the primary meridional angle to bring the meridian to 180° prior to computation. Following computation, the system can be ‘un-rotated’ to return results to the original coordinate system. When analysing using the user-defined surface in Zemax, the coordinate break surface type can be applied to effect such a rotation of the meridians.

It should also be noted that the meridian mapping equations (Eqs. (1)–(6), also see Appendix) require *β* to be bounded within the open interval of 0° and 180°. That is, *β* cannot take on a value of either 0° or 180°, as that would create a surface for which the rate of change of meridional curvature with meridional angle is infinite.

## Conclusion

Unlike conventional, or orthogonal, ophthalmic lenses, non-orthogonal lenses have principal meridians that are not perpendicular to each other. A modification to conventional sphero-cylindrical transposition has been proposed for these lenses, and a procedure has been determined to allow raytracing with them. The next step is to determine whether the use of a bi-cubic non-orthogonal surface can improve retinal image quality, theoretically at least, for people with irregular astigmatism, such as occurs in keratoconus.

## Supplementary information

Supplementary video

Supplementary information

## Figures and Tables

**Fig. 10:**
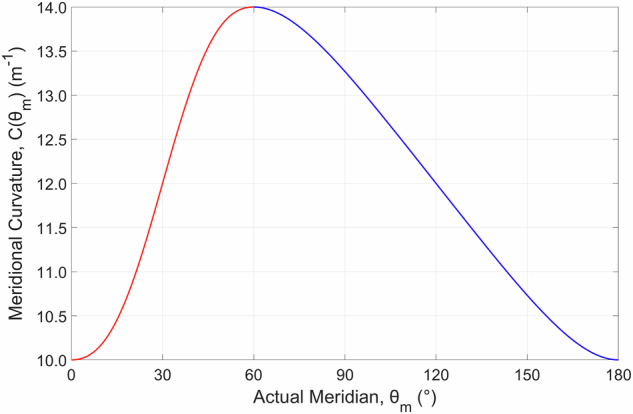
Meridional curvature calculated from the bi-cubic function example of Fig. 3 for the case +5.00/+2.00 (60)×180, showing the curvature from the lower sub-function (red) and upper sub-function (blue).

## Data Availability

No datasets were generated or analysed during the current study.

## Appendix

**Background**

For a conventional cylinder surface, the meridional curvature can be given as:

A1 $$C({\theta }_{{{m}}})={C}_{{{\rm{p}}}}+({C}_{{{\rm{s}}}}-{C}_{{{\rm{p}}}}){\sin }^{2}({\theta }_{{{m}}})$$

*θ*_m_ is the meridional angle in degrees,

*C* is the meridional curvature in m^–1^,

*C*_p_ is the primary meridional curvature of the cylinder in m^–1^, and

*C*_s_ is the secondary meridional curvature of the cylinder in m^–1^.

Equation (A1) can be restated as a mapping of the actual, physical meridional ${\theta }_{{{m}}}$ on the surface to a curvature-defining meridional angle ${\theta }_{{{\rm{e}}}}$ (Fig. 10). That is,

A2 $$C({\theta }_{{{m}}})={C}_{{{\rm{p}}}}+({C}_{{{\rm{s}}}}-{C}_{{{\rm{p}}}}){\sin }^{2}({\theta }_{{{\rm{e}}}})$$

where ${\theta }_{{{\rm{e}}}}$ is some function of ${\theta }_{{{m}}}$

A3 $${\theta }_{{{\rm{e}}}}=F({\theta }_{{{m}}})$$

Trivially, for a conventional cylinder

$${\theta }_{{{\rm{e}}}}={\theta }_{{{m}}}$$

**Bi-Cubic Meridional Mapping**

To minimise complexity, the angle of the primary power meridional angle ${\theta }_{{{\rm{p}}}}$ is assumed to be 0°/180° for the remainder of this derivation. In addition, in the following derivation values for the actual meridional angle ${\theta }_{{{\rm{p}}}}$ and curvature-determining angle ${\theta }_{{{\rm{e}}}}$ are operated as their values modulo 180°. For example, if the actual meridional angle is 225°, this angle modulo 180° is the remainder of 225°/180°, that is, 45°, which is the value that is used for calculations.

In the non-orthogonal cylinder surface of the present study, $F({\theta }_{{{m}}})$ of Eq. (A3) is defined as a “bi-cubic” piece-wise smooth function consisting of lower and upper sub-functions, acting on the sector intervals ${0}^{\circ } < {\theta }_{{{m}}}\le {\theta }_{{{\rm{s}}}}$ and ${\theta }_{{{\rm{s}}}} < {\theta }_{{{m}}}\le {180}^{\circ }$, respectively, where ${\theta }_{{{\rm{s}}}}$ is the angle of the secondary power meridian.

The sub-functions are third-order polynomials relating ${\theta }_{{{\rm{e}}}}$ to ${\theta }_{{{m}}}$:

A4 $${\theta }_{{{\rm{e}}}}=\displaystyle {\sum }_{k=0}^{3}{b}_{k}{\theta }_{{{m}}}^{k}$$

where

*k* is the order, and

*b*_*k*_ is the *k*th polynomial coefficient.

A smooth non-orthogonal cylinder surface requires a smooth transition of meridional curvature with meridional angle. To satisfy this requirement, two constraints are imposed on the sub-functions. Firstly, the sub-functions must meet at a common value for ${\theta }_{{{\rm{e}}}}$. Secondly, the sub-functions must meet with common tangents of unit slope, i.e., ${{d}}{\theta }_{{{\rm{e}}}}/{{d}}{\theta }_{{{m}}}=1$ at their meeting points. Applying these constraints to Eq. (A4) results in the following equations for the polynomial coefficients:

A5 $$\left.\begin{array}{rc}\left(\begin{array}{rc}{b}_{0}=0\hfill\\ {b}_{1}=1\hfill \\ {b}_{2}=\frac{540-6{\theta }_{{{\rm{s}}}}}{2{\theta }_{{{\rm{s}}}}^{2}}&\qquad\qquad\qquad\qquad{{for}}\,{0}^{\circ } \, < \, {\theta }_{{m}}\le {\theta }_{{{\rm{s}}}} \\ {b}_{3}=\frac{2{\theta }_{{{\rm{s}}}}-180}{{\theta }_{{{\rm{s}}}}^{3}}\end{array} \right. \\ \left(\begin{array}{cc}{b}_{0}=\frac{97200({\theta }_{{{\rm{s}}}}-60)(90-{\theta }_{s})}{{({\theta }_{{{\rm{s}}}}-180)}^{3}}\\ {b}_{1}=\frac{{\theta }_{{{\rm{s}}}}^{3}+540{\theta }_{{{\rm{s}}}}^{2}-5832000}{{({\theta }_{{{\rm{s}}}}-180)}^{3}}\\ {b}_{2}=\frac{-3({\theta }_{{{\rm{s}}}}+180)({\theta }_{{{\rm{s}}}}-90)} {({\theta }_{{{\rm{s}}}}-180)^{3}}&\qquad\qquad{{for}}\,{{\theta }_{{{\rm{s}}}} \, < \, {\theta }_{{m}} \le {180}^{\circ }}\\ {b}_{3}=\frac{2{\theta }_{{{\rm{s}}}}-180}{({\theta }_{{{\rm{s}}}}-180)^{3}}\hfill\end{array} \right.\end{array}\right\}$$

Equation A4 and A5 give the curvature-determining meridional angle ${\theta }_{{{\rm{e}}}}$ for a given actual meridional angle ${\theta }_{{{m}}}$ (Fig. 3). That is, the meridional curvature along ${\theta }_{{{m}}}$ on the non-orthogonal cylinder will be the same as that along ${\theta }_{{{\rm{e}}}}$ for a conventional cylinder. Combining Eqs. (A2), (A4) and (A5), and given the meridional curvatures of the principal meridians ${C}_{{{\rm{p}}}}$ and ${C}_{{{\rm{s}}}}$, and their meridional angles ${\theta }_{{{\rm{p}}}}$ and ${\theta }_{{{\rm{s}}}}$, the meridional curvature $C({\theta }_{{{m}}})$ for the non-orthogonal cylinder surface can be calculated (Fig. A1).

**Surface Sag**

Given the circumferential set of meridional curvatures, the surface sag for the non-orthogonal cylinder can be calculated by the equation:

A6 $$z({\theta }_{{{m}}},r)=\frac{({C}_{{{\rm{p}}}}+({C}_{{{\rm{s}}}}-{C}_{{{\rm{p}}}}){\sin }^{2}({\theta }_{{{\rm{e}}}})){r}^{2}}{1+\sqrt{1-{({C}_{{{\rm{p}}}}+({C}_{{{\rm{s}}}}-{C}_{{{\rm{p}}}}){\sin }^{2}({\theta }_{{{\rm{e}}}}))}^{2}{r}^{2}}}$$

where

*z* is the surface sag in metres,

*r* is the radial position of the surface point in metres, and

*θ*_e_ is calculable using Eqs. (A4) and (A5)

**Partial Derivatives**

For raytracing, the surface normal at the point of refraction is required. This can be calculated from the partial derivatives of Eq. (A6) for surface sag, which in cylindrical coordinates are:

A7 $$\frac{{{\partial }}z}{{{\partial }}{\theta }_{{{m}}}}=	\frac{2D\frac{{{d}}{\theta }_{{{\rm{e}}}}}{{{d}}{\theta }_{{{m}}}}\sin ({\theta }_{{{\rm{e}}}})\cos ({\theta }_{{{\rm{e}}}}){r}^{2}}{1+\sqrt{1-{({C}_{{{\rm{p}}}}+D{\sin }^{2}({\theta }_{{{\rm{e}}}}))}^{2}{r}^{2}}} \\ +	 \frac{2D{({C}_{{{\rm{p}}}}+D{\sin }^{2}({\theta }_{{{\rm{e}}}}))}^{2}{r}^{4}\frac{{{d}}{\theta }_{{{\rm{e}}}}}{{{d}}{\theta }_{{{m}}}}\sin ({\theta }_{{{\rm{e}}}})\cos ({\theta }_{{{\rm{e}}}})}{{(1+\sqrt{1-{({C}_{{{\rm{p}}}}+D{\sin }^{2}({\theta }_{{{\rm{e}}}}))}^{2}{r}^{2}})}^{2}\sqrt{1-{({C}_{{{\rm{p}}}}+D{\sin }^{2}({\theta }_{{{\rm{e}}}}))}^{2}{r}^{2}}}$$

A8 $$\frac{{{\partial }}z}{{{\partial }}r}	=\frac{2{({C}_{{{\rm{p}}}}+D{\sin }^{2}({\theta }_{{{\rm{e}}}}))}^{2}r}{1+\sqrt{1-{({C}_{{{\rm{p}}}}+D{\sin }^{2}({\theta }_{{{\rm{e}}}}))}^{2}{r}^{2}}}\\ 	+\frac{{({C}_{{{\rm{p}}}}+D{\sin }^{2}({\theta }_{{{\rm{e}}}}))}^{3}{r}^{3}}{{(1+\sqrt{1-{({C}_{{{\rm{p}}}}+D{\sin }^{2}({\theta }_{{{\rm{e}}}}))}^{2}{r}^{2}})}^{2}\sqrt{1-{({C}_{{{\rm{p}}}}+D{\sin }^{2}({\theta }_{{{\rm{e}}}}))}^{2}{r}^{2}}}$$

where

$${D=C}_{{{\rm{s}}}}-{C}_{{{\rm{p}}}}$$, and

$$\frac{{{d}}{\theta }_{{{\rm{e}}}}}{{{d}}{\theta }_{{{m}}}}=\displaystyle {\sum }_{j=1}^{3}{b}_{j}j{\theta }_{{{m}}}^{(j-1)}$$

**Cross Derivative**

For modelling the non-orthogonal cylinder using surface fitting techniques (e.g., the grid-sag surface of Ansys Zemax OpticStudio), having the cross-derivative for surface sag can provide smoother interpolation between grid points. In cylindrical coordinates, the cross-derivative is

A9 $$\frac{{{{\partial }}}^{2}z}{{{\partial }}{\theta }_{{{m}}}{{\partial }}r}	 = \frac{4D\frac{{{d}}{\theta }_{{{\rm{e}}}}}{{{d}}{\theta }_{{{m}}}}\sin ({\theta }_{{{\rm{e}}}})\cos ({\theta }_{{{\rm{e}}}})r}{1+\sqrt{1-{({C}_{{{\rm{p}}}}+D{\sin }^{2}({\theta }_{{{\rm{e}}}}))}^{2}{r}^{2}}}\\ 	+\frac{10{D({C}_{{{\rm{p}}}}+D{\sin }^{2}({\theta }_{{{\rm{e}}}}))}^{2}{r}^{3}\frac{{{d}}{\theta }_{{{\rm{e}}}}}{{{d}}{\theta }_{{{m}}}}\sin ({\theta }_{{{\rm{e}}}})\cos ({\theta }_{{{\rm{e}}}})}{{(1+\sqrt{1-{({C}_{{{\rm{p}}}}+D{\sin }^{2}({\theta }_{{{\rm{e}}}}))}^{2}{r}^{2}})}^{2}\sqrt{1-{({C}_{{{\rm{p}}}}+D{\sin }^{2}({\theta }_{{{\rm{e}}}}))}^{2}{r}^{2}}}\\ 	+\frac{4{D({C}_{{{\rm{p}}}} +D{\sin }^{2}({\theta }_{{{\rm{e}}}}))}^{4}{r}^{5}\frac{{{d}}{\theta }_{{{\rm{e}}}}}{{{d}}{\theta }_{{{m}}}}\sin ({\theta }_{{{\rm{e}}}})\cos ({\theta }_{{{\rm{e}}}})}{{(1+\sqrt{1-{({C}_{{{\rm{p}}}}+D{\sin }^{2}({\theta }_{{{\rm{e}}}}))}^{2}{r}^{2}})}^{3}(1-{({C}_{{{\rm{p}}}}+D{\sin }^{2}({\theta }_{{{\rm{e}}}}))}^{2}{r}^{2})}\\ 	+\frac{2{D({C}_{{{\rm{p}}}}+D{\sin }^{2}({\theta }_{{{\rm{e}}}}))}^{4}{r}^{5}\frac{d{\theta }_{{\rm{e}}}}{{{d}}{\theta }_{{{m}}}}\sin ({\theta }_{{{\rm{e}}}})\cos ({\theta }_{{{\rm{e}}}})}{{(1+\sqrt{1-{({C}_{{{\rm{p}}}}+D{\sin }^{2}({\theta }_{{{\rm{e}}}}))}^{2}{r}^{2}})}^{2}\sqrt{{(1-{({C}_{{{\rm{p}}}}+D{\sin }^{2}({\theta }_{{{\rm{e}}}}))}^{2}{r}^{2})}^{3}}}$$

**Technical Note**

The above equations were derived assuming rotation of the principal meridians so that ${\theta }_{{{\rm{p}}}}$ is 0° (or 180°). To calculate meridional curvatures for cases where ${\theta }_{{{\rm{p}}}}$ is non-zero, ${\theta }_{{{\rm{p}}}}$ (modulo 180°) is subtracted from all angles, but added back after applying the equations.
