# Supplementary material for: Principles of a Non-orthogonal Optical Surface with Potential for Correction of Irregular Astigmatism
Source: Ophthalmic Physiol Opt. 2026 Apr 15;46(3):502–9. doi: 10.1007/s44402-026-00068-6 (PMC13369654; doi:10.1007/s44402-026-00068-6)
Supplement: Supplementary file 2 — Supplementary information [file 44402_2026_68_MOESM2_ESM.docx]

**Supplementary file**

The video ah_noc_02_TF.avi shows through-focus spot diagrams for the non-orthogonal lens between 142 mm and 200 mm focal lines (scale on side 800 μm).
